# A Family Segregating Lethal Primary Coenzyme Q10 Deficiency Due to Two Novel *COQ6* Variants

**DOI:** 10.3389/fgene.2021.811833

**Published:** 2022-01-17

**Authors:** Na Wang, Youmin Zheng, Lingzi Zhang, Xiong Tian, Yicheng Fang, Ming Qi, Juping Du, Shuaishuai Chen, Shiyong Chen, Jun Li, Bo Shen, Lizhen Wang

**Affiliations:** ^1^ Department of Clinical Laboratory, Taizhou Hospital of Zhejiang Province Affiliated to Wenzhou Medical University, Linhai, China; ^2^ Department of Pediatrics, Taizhou Hospital of Zhejiang Province Affiliated to Wenzhou Medical University, Linhai, China; ^3^ Department of Public Research Platform, Taizhou Hospital of Zhejiang Province Affiliated to Wenzhou Medical University, Linhai, China; ^4^ Department of Radiology, Taizhou Hospital of Zhejiang Province Affiliated to Wenzhou Medical University, Linhai, China; ^5^ Sir Run Run Shaw Hospital Affiliated to Zhejiang University School of Medicine, Hangzhou, China; ^6^ DIAN Diagnostics, Hangzhou, China; ^7^ Department of Pathology and Laboratory Medicine, University of Rochester Medical Center, Rochester, NY, United States

**Keywords:** *COQ6* mutation, nephrotic syndrome, primary coenzyme Q10 deficiency, genetics, infancy

## Abstract

Primary coenzyme Q10 deficiency-6 (COQ10D6), as a rare autosomal recessive disease caused by *COQ6* mutations, is characterized by progressive infantile-onset nephrotic syndrome resulting in end-stage renal failure and sensorineural hearing loss. Here, we report two Chinese siblings with COQ10D6 who primarily presented with severe metabolic acidosis, proteinuria, hypoalbuminemia, growth retardation, and muscle hypotonia and died in early infancy. Using whole-exome sequencing and Sanger sequencing, we identified two rare recessive nonsense mutations in the *COQ6* gene segregating with disease in affected family members: c.249C > G (p.Tyr83Ter) and c.1381C > T (p.Gln461Ter), resulting in two truncated protein products. Both mutations are located in a highly conserved area and are predicted to be pathogenic. Indeed, the death of our patients in early infancy indicates the pathogenicity of the p.Tyr83Ter and p.Gln461Ter variants and highlights the significance of the two variants for *COQ6* enzyme function, which is necessary for the biosynthesis of coenzyme Q10. In conclusion, we discovered a novel compound heterozygous pathogenic variant of the *COQ6* gene as a cause of severe COQ10D6 in the two siblings. Based on the clinical history and genetic characteristics of the patients, our cases expand the genotypic spectrum of COQ10D6 and highlight the heterogeneity and severity of clinical features associated with *COQ6* mutations. For patients with clinical manifestations suggestive of COQ10D6, early testing for *COQ6* mutations is beneficial for disease diagnosis and therapeutic interventions as well as disease prevention in future generations.

## Introduction

Nephrotic syndrome (NS) is a chronic kidney disease that involves massive proteinuria, hypoalbuminemia, hyperlipidemia, and edema. When occurring in the first year of life, NS is considered a life-threatening clinical condition with poor prognosis and high mortality if not treated in time (Noone et al., 2018). NS is generally classified as steroid-dependent nephrotic syndrome (SDNS), steroid-resistant nephrotic syndrome (SRNS), or steroid-sensitive nephrotic syndrome (SSNS) based on its response to steroid therapy (Greenbaum et al., 2012; Trautmann et al., 2017). SRNS is a common cause of end-stage renal disease (ESRD) in children, affecting approximately 10–20% of all pediatric NS cases (Warejko et al., 2018; Park et al., 2020), and the renal biopsies of affected patients usually show focal segmental glomerulosclerosis (FSGS) (Lovric et al., 2016).

Recently, large cohort studies have shown that approximately 30% of childhood-onset SRNS cases are associated with genetic defects (Sadowski et al., 2015; Wang et al., 2017; Nagano et al., 2020). Different variants of the genes involved in coenzyme Q10 (CoQ10) biosynthesis may lead to a renal phenotype, either syndromic SRNS (*PDSS2*, *COQ2*, and *COQ6*) or isolated SRNS (*COQ8B*) (Cheong 2020). Primary CoQ10 deficiency-6 (COQ10D6, OMIM # 614650) is an autosomal recessive disorder that manifests as severe progressive infantile-onset NS resulting in end-stage renal failure and sensorineural hearing loss (SNHL) due to homozygous or compound heterozygous mutations in the *COQ6* gene located on chromosome 14q24.3, which encodes an evolutionarily conserved flavin-dependent monooxygenase required for CoQ10 biosynthesis. Heeringa et al. (Heeringa et al., 2011) first reported that 11 children from five families with COQ10D6 manifest as NS and SNHL, and some of these patients responded favorably to oral CoQ10 treatment. In addition, high-dose exogenous CoQ10 supplementation at early stages of the disease can ameliorate the neurological and renal symptoms (Heeringa et al., 2011; Quinzii et al., 2014; Stanczyk et al., 2018). Nevertheless, patients may not benefit from CoQ10 therapy when severe renal and neurological damage is established. Hence, an early and accurate diagnosis of COQ10D6 and simultaneous CoQ10 intervention are critical in improving prognosis.

In this study, we identified a novel compound heterozygous variant of the *COQ6* gene in a nonconsanguineous Chinese family with two siblings presenting severe metabolic acidosis, proteinuria, hypoalbuminemia, growth retardation, and muscle hypotonia; the female proband also developed seizures. Genetic findings showed that both mutations, located in the Ubi-OHase domain of *COQ6*, are present in a region that is highly evolutionarily conserved across species. The mutations are predicted to be pathogenic, as confirmed by the death of our patients in early infancy. Collectively, our findings widen the spectrum of known *COQ6* mutations and provide an expanded understanding of the clinical spectrum of the rare genetic disease COQ10D6.

## Results

### Clinical Presentations of the Family

**FIGURE 1:**
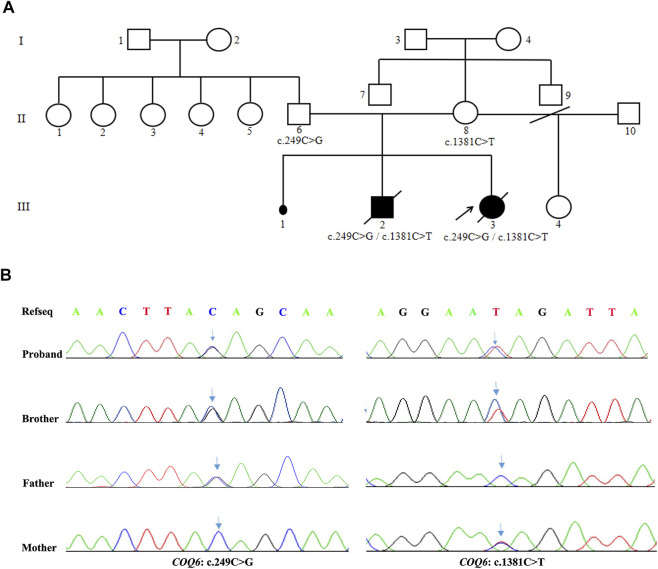
Family pedigree and confirmation of the compound heterozygous variants of *COQ6*. **(A)** Pedigree shows the cosegregation of *COQ6* mutations in the family. The patient pointed by the arrow indicates the proband. Black symbols indicate clinically affected members, and open symbols represent unaffected individuals. **(B)** Validation of *COQ6* variants in the family by Sanger sequencing, and the arrows represent sites of mutation.

The two affected siblings were conceived by a healthy, nonconsanguineous couple who denied having hepatitis, tuberculosis, diabetes, allergic diseases, or a history of hereditary disease. The mother of the patients has a history of miscarriages. A summary of the molecular findings and clinical and biochemical characteristics of the two cases harboring *COQ6* mutations is shown in Table 1. The pedigree of the family and the confirmation of the *COQ6* mutations are presented in Figure 1A.

**TABLE 1:** Clinical characteristics and molecular findings in subjects with COQ6 mutations.

	Proband (III-3)	Sibling of proband (III-2)
*COQ6* mutation	c.249C > G/c.1381C > T	c.249C > G/c.1381C > T
Sex	Female	Male
Pregnancy duration	Full term	Full term
Type of delivery	Spontaneous vaginal delivery	Spontaneous vaginal delivery
Age at presentation	4 months + 23 days	3 months + 22 days
Birth Weight (kg)	3.1	2.95
Birth length (cm)	50	50
Feeding difficulties	–	–
Respiratory distress	+	+
Muscle hypotonia	+	+
Seizure	+	–
SNHL	–	–
Edema	+	+
Lactate (mmol/L)	5.4	7.8
Proteinuria (mg/24 h)	2,570	n/a
Serum albumin(g/L)	23.7	24.8
UOA	Lactic acid-2, pyruvic acid-OX-2, 3-hydroxybutyric acid-2, palmitic acid-1, 4-hydroxy-phenyllactate-2, malic acid-3	n/a
Brain MRI or head CT	Bifrontal widening frontotemporal of the subarachnoid space; delayed myelination of white matter	Bifrontal widening frontotemporal of the subarachnoid space
Age at death	5 months + 26 days	4 months + 13 days

Note: SNHL, sensorineural hearing loss; UOA, urine organic acid; MRI, magnetic resonance imaging; CT, computed tomography; n/a, not done or not available.

### Patient 1

**FIGURE 2:**
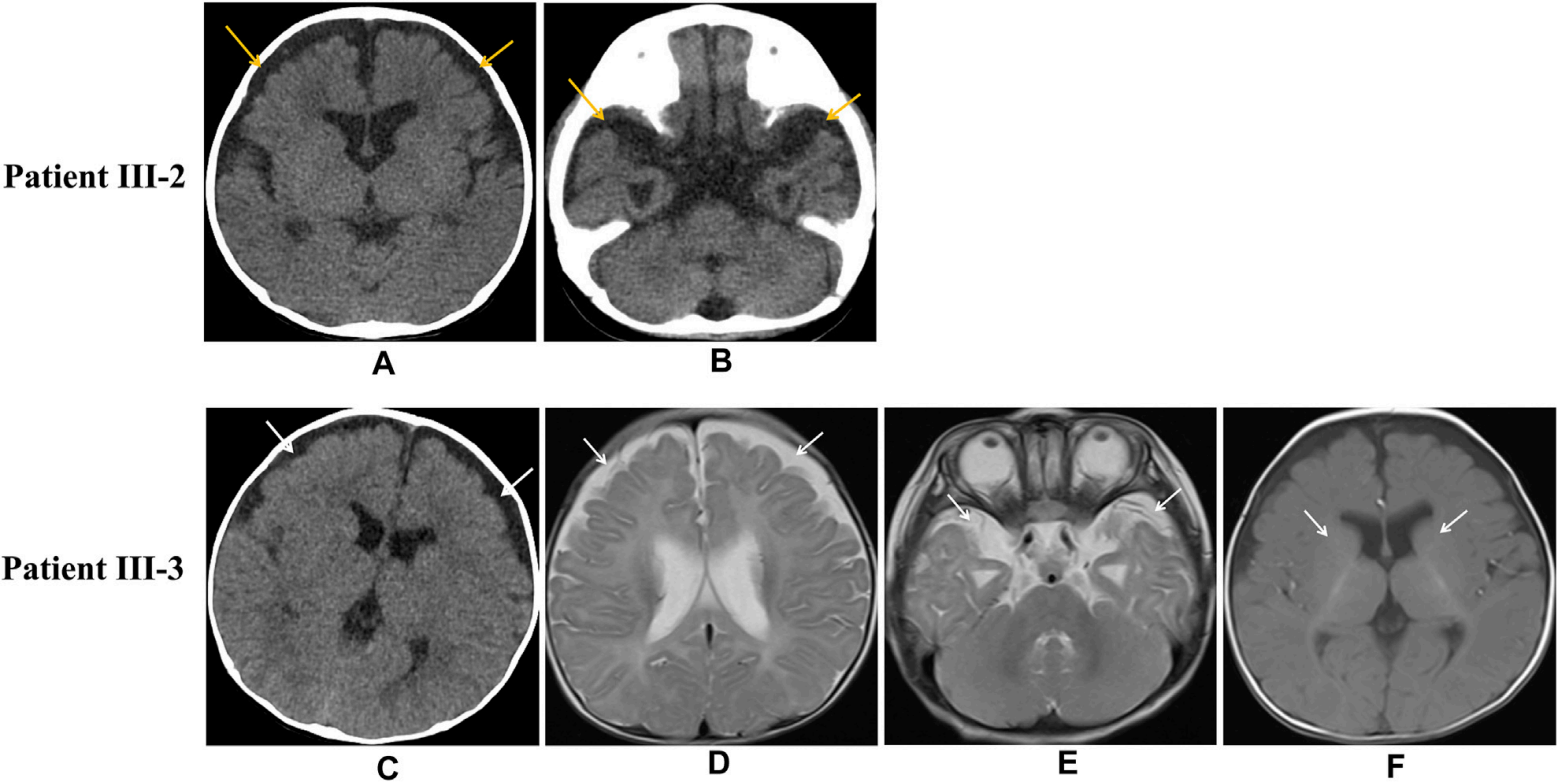
Imaging of patient III-2 and patient III-3. Unenhanced head CT indicates widening of the bifrontal frontotemporal subarachnoid space in patient III-2 (Figures 2A,B) Unenhanced head CT of patient III-3 shows bifrontal widening frontotemporal of the subarachnoid space (Figure 2C), and axial MRI shows bifrontal widening frontotemporal of the subarachnoid space (Figures 2D,E) and delayed myelination of white matter (Figure 2F).

The proband (III-3) was a Chinese girl born at full term *via* spontaneous vaginal delivery who weighed 3.1 kg. According to her parents, the infant had not achieved the expected increases in height and body weight by the age of 3 months. At the age of 4 months and 28 days, she was sent to our hospital due to repeated convulsion for 5 days. Metabolic and hearing exams were normal. The blood gas analysis indicated compensatory metabolic acidosis with an elevated lactate level (5.4 mmol/L), a pH of 7.39, and a bicarbonate level of 13.4 mmol/L. Biochemical assays revealed significantly decreased albumin (23.7 g/L) and increased blood lipids (triglyceride (TG), 15.80 mmol/L; total cholesterol (TC), 9.54 mmol/L). A decline in immunity was also observed with IgG <0.75 g/L, IgA <0.01 g/L, and IgM <0.20 g/L. The urine organic acid (UOA) analysis showed increased levels of lactate, 3-hydroxybutyric acid-2, pyruvic acid-OX-2, 4-hydroxy-phenyllactate-2, palmitic acid-1, and malic acid-3. A widening of the bilateral frontotemporal subarachnoid space was detected by enhanced computed tomography (CT) (Figure 2C), though her electroencephalogram (EEG) was normal. During hospitalization, the patient developed a seizure during which her hands were clenched; the seizure abated after half a minute. The girl was then transferred to a higher-level hospital at the request of her parents. Magnetic resonance imaging (MRI) demonstrated a widening of the bilateral frontotemporal subarachnoid space (Figures 2D,E) and delayed myelination of white matter (Figure 2F), but long-term EEG showed no abnormality. Laboratory testing indicated a markedly elevated TC level of 9.79 mmol/L, an IgG level of 0.1 g/L, a CD19^+^ B cell percentage of 14.7%, a CD3^+^ T-cell percentage of 72.8%, and a CD4^+^ T-cell percentage of 50.3%. The girl’s parents declined further genetic testing, and she was discharged home the next day.

At the age of 5 months and 22 days, she was admitted again to the Department of Pediatrics of our hospital because of pneumonia and dyspnea. Physical examination indicated that she had growth retardation, with a height of 54 cm and body weight of 5.5 kg, which were both below −3 SD, and she was unable to raise her head steadily. Laboratory tests revealed hypoalbuminemia (16 g/L), massive proteinuria (3+, total urine protein of 2,570 mg/24 h), edema, and elevated blood lipids (TG, 27.51 mmol/L; TC, 13.77 mmol/L), accompanied by metabolic acidosis and electrolyte disturbances including decreased Na^+^ (119 mmol/L) and elevated K^+^ (6.48 mmol/L). After admission, the patient received anti-infection and symptomatic treatment including repeated albumin for protein supplementation and repeated acid correction. Given the proband’s clinical symptoms and age of onset, NS was considered and a blood sample was then collected for genetic testing. Unfortunately, she had persistent metabolic acidosis, hyponatremia, and hypoalbuminemia. She continued to deteriorate and died of respiratory failure and heart failure at the age of 5 months and 26 days.

### Patient 2

Patient III-2 was the brother of the proband. He was also born at full term *via* spontaneous vaginal delivery with a birth weight of 2.95 kg. At the age of 3 months and 22 days, he was admitted to our hospital because of repeated abdominal distension for 3 months, along with dyspnea and cyanosis for half an hour. Upon hospitalization, physical examination was unremarkable except for dyspnea and poor growth and development, with a body weight of 4.5 kg and a height of 57 cm, which were both below −2 SD. Laboratory examination showed cytomegalovirus infection, proteinuria (2+), and occult blood (2+). Blood gas analysis indicated compensatory respiratory alkalosis combined with metabolic acidosis with lactate at 7.8 mmol/L. Moreover, he exhibited significant decreases in albumin (24.8 g/L) and total protein (40.7 g/L), along with an IgA level of 0.02 g/L and increased lactic acid dehydrogenase (1123 U/L) and TG (6.02 mmol/L). Cardiac ultrasound revealed an atrial septal defect and pulmonary hypertension, and an electrocardiogram showed altered sinus rhythm T waves. The patient did not undergo a metabolic study, but his neonatal screening was normal. Regrettably, the boy remained in a critical condition after the application of symptomatic treatment.

The patient was then transferred to a higher-level hospital at the request of the parents. Chest and abdominal CT detected pulmonary inflammation and slightly enlarged liver, and abdominal ultrasound showed enlarged kidneys and poor liver texture. A widening of the bifrontal frontotemporal region of the subarachnoid space was indicated by the cranial CT exhibited (Figures 2A,B). Meanwhile, laboratory tests showed hypoalbuminemia, proteinuria (2+), and edema as well as a decreased level of IgG (2.2 g/L). However, hypoalbuminemia was not ameliorated by symptomatic treatment, and he subsequently developed exacerbated lung infection and degressive transcutaneous oxygen saturation. Unfortunately, despite all treatments, he died due to pulmonary hemorrhage and respiratory failure at the age of 4 months and 13 days.

### Genetic Findings

The proband (III-3) underwent genetic testing by WES. DNA samples from her parents and brother were tested to verify the variants using Sanger sequencing. As illustrated in Figure 1B, novel compound heterozygous mutations in the *COQ6* gene (NM_182476.3:exon2:c.249C > G (p.Tyr83Ter) and exon12:c.1381C > T (p.Gln461Ter)) were found in the proband (III-3) and her brother (III-2), and Sanger sequencing validation identified their parents as heterozygous carriers for one of the mutations.

**FIGURE 3:**
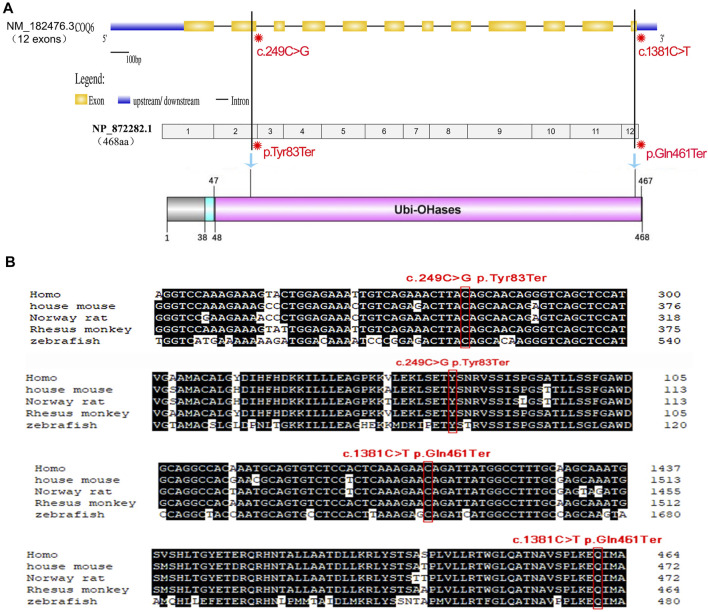
Schematic of the gene structure and domain structure of *COQ6* protein and conservation analysis of the two nonsense mutations detected in the family. **(A)** The *COQ6* gene containing 12 exons (yellow boxes) was exhibited using the UDSD online database, and the location of the two variants is tagged in the *COQ6* exon region and its corresponding Ubi-OHases domain. **(B)** Comparative analysis of genome sequences or CDSs across species indicates that the two mutant sites are evolutionarily highly conserved.

Figure 3A shows a schematic of the *COQ6* gene containing the two identified variants, and the COQ6 protein with its functional domains was created. The first variant c.249C > G (p.Tyr83Ter) in exon 2 of 12 causes a change of amino acid 83 from tyrosine to a stop codon that results in a truncated protein or degradation and thus probably greatly affects the structure and function of the *COQ6* enzyme. *COQ6* variant c.1381C > T (p.Gln461Ter) occurs at the last exon and leads to a deletion of seven amino acids from the Ubi-OHases domain of *COQ6*. Evolutionarily, a tyrosine at position 83 and a glutamine at position 461 of the *COQ6* enzyme are highly conserved among various species (Figure 3B). According to the gnomAD database, these variants are detected with no homozygosity at allele frequencies of 0.00003185 and 0.00003188, demonstrating that they are not common benign variants in the populations included in the database. Furthermore, both variants are absent from ClinVar, and no previous patient with either variant has been described in the literature to date. Using MutationTaster, p.Tyr83Ter and p.Gln461Ter are predicted to be disease-causing variants and p.Tyr83Ter may lead to nonsense-mediated decay (NMD).

## Discussion

In the present study, we report two novel pathogenic *COQ6* mutations: c.249C > G (p.Tyr83Ter) and c.1381C > T (p.Gln461Ter), which, in a compound heterozygous state, resulted in two siblings with clinical presentations of COQ10D6 characterized by renal involvements including hypoalbuminemia, proteinuria and edema, and extra-renal manifestations including growth retardation, muscle hypotonia, and/or seizures. Both mutations cosegregated with the disease, and the pathogenicity of the two *COQ6* variants was confirmed by the clinical symptoms of our patients, the analysis of human genome variation databases, and *in silico* predictions.

CoQ10, also known as ubiquinone, is an important component of the mitochondrial electron transport chain; this compound exerts many effects, such as countering oxidation, regulating calcium homeostasis, improving mitochondrial function, and preventing cellular apoptosis (Hargreaves et al., 2020). To date, pathogenic mutations in 10 genes (*PDSS1*, *PDSS2*, *COQ2*, *COQ4*, *COQ6*, *COQ7*, *COQ8A/ADCK3*, *COQ8B/ADCK4,* and *COQ9*) involved in the biosynthesis of CoQ10 have been reported to cause primary CoQ10 deficiency, which has variable clinical symptoms ranging from fatal neonatal multisystem disorder to adult-onset encephalopathy or nephropathy (Desbats et al., 2015; Acosta et al., 2016). Several pathogenic mutations in the *COQ6* gene are known to cause kidney dysfunction such as SRNS and extrarenal manifestations such as SHNL, seizures, growth retardation, and mild muscle weakness in the lower extremities (Heeringa et al., 2011; Cao et al., 2014; Park et al., 2017). The clinical manifestations in our cases and other reported patients are summarized in Table 2.

**TABLE 2:** Genotypic and phenotypic characteristics of the detected *COQ6* variants in the published literature and the present study.

Origin	Age at onset (year)	Age at ESRD (year)	*COQ6* mutations	Zygosity	Renal involvement	Extra-renal involvement	Effect of CoQ10 and its analogue treatment	References
China	0.4	No	c.249C > G (p.Tyr83Ter)	Heterozygous	NS	Growth retardation, seizures, muscle hypotonia	ND	Current study
			c.1381C > T (p.Gln461Ter)					
China	0.3	No	c.249C > G (p.Tyr83Ter)	Heterozygous	Proteinuria	Growth retardation, muscle hypotonia	ND	Current study
			c.1381C > T (p.Gln461Ter)					
Turkey	0.2	NA	c.763G > A (p.Gln255Arg)	Homozygous	SRNS	SNHL, bilateral nephrolithiasis, growth retardation	Recovery of kidney function	Heeringa et al. (2011)
Turkey	0.3	0.4	c.763G > A (p.Gln255Arg)	Homozygous	SRNS	SNHL, facial dysmorphism	SNHL substantially improved	Heeringa et al. (2011)
Turkey	0.3	0.4	c.763G > A (p.Gln255Arg)	Homozygous	SRNS	Seizures	ND	Heeringa et al. (2011)
Lebanon	0.3	1.7	c.763G > A (p.Gln255Arg)	Homozygous	SRNS	SNHL	ND	Heeringa et al. (2011)
Lebanon	<1.0	3.0	c.763G > A (p.Gln255Arg)	Homozygous	SRNS	SNHL	ND	Heeringa et al. (2011)
Lebanon	1.2	1.4	c.763G > A (p.Gln255Arg)	Homozygous	SRNS	SNHL, ataxia	ND	Heeringa et al. (2011)
Lebanon	6.4	9.3	c.763G > A (p.Gln255Arg)	Homozygous	SRNS	SNHL	ND	Heeringa et al. (2011)
Turkey	2.5	3.4	c.1058C > A (p.Ala353Asp)	Homozygous	SRNS	SNHL, seizures, white matter abnormalities	ND	Heeringa et al. (2011)
Turkey	6.0	6.5	c.1058C > A (p.Ala353Asp)	Homozygous	SRNS	SNHL	ND	Heeringa et al. (2011)
Turkey	2.5	NA	c.1058C > A (p.Ala353Asp)	Homozygous	SRNS	SNHL	Remission of proteinuria	Heeringa et al. (2011)
Turkey	3.0	NA	c.1341G > A (p.Trp447Ter)	Heterozygous	SRNS	SNHL	ND	Heeringa et al. (2011)
			c.1383delG (p.Gln461fsTer478)					
European	4.5	NA	c.1154A > C (p.Asp385Ala)	Heterozygous	SRNS	NA	ND	Sadowski et al. (2015)
			c.1235A > G (p.Tyr412Cys)					
China	0.8	NA	c.1078C > T (p.R360W)	Homozygous	SRNS	SNHL, growth retardation, muscle hypotonia	Complete remission of NS; improved psychomotor development	Cao et al. (2017)
Korea	1.2	1.4	c.189_191delGAA (p.Lys64del); c.782C > T (p.Pro261Leu)	Heterozygous	SRNS	SNHL, bilateral optic nerve atrophy	NA	Park et al. (2017)
Korea	1.9	2.6	c.189_191delGAA (p.Lys64del); c.686A > C (p.Gln229Pro)	Heterozygous	SRNS	SNHL, extropia with nistagmus on both eyes	NA	Park et al. (2017)
Korea	2.0	3.6	c.189_191delGAA (p.Lys64del); c.782C > T (p.Pro261Leu)	Heterozygous	SRNS	SNHL, mild muscle weakness in the lower extremities	NA	Park et al. (2017)
Korea	2.6	4.6	c.189_191delGAA (p. Lys64del); c.782C > T (p.Pro261Leu)	Heterozygous	SRNS	SNHL	NA	Park et al. (2017)
Korea	3.8	6.1	c.189_191delGAA (p. Lys64del); c.782C > T (p.Pro261Leu)	Heterozygous	SRNS	SNHL, mild muscle weakness in the lower extremities	NA	Park et al. (2017)
Korea	3.9	4.0	c.189_191delGAA (p. Lys64del); c.782C > T (p.Pro261Leu)	Heterozygous	SRNS	SNHL	NA	Park et al. (2017)
Italy	0.6	1.7	c.782C > T (p.Pro261Leu)	Homozygous	SRNS	None	Delayed neurological disease	Gigante et al. (2017)
China	16	NA	c.41G > A (p.Try14Ter)	Homozygous	SRNS	None	ND	Song et al. (2018)
China	0.2	NA	c.1078C > T (p.R360W)	Homozygous	Congenital NS	None	ND	Li et al. (2018)
Poland	2.0	NA	c.1078C > T (p. Arg360Trp); c.804delC (p.Leu269TrpfsTer13)	Heterozygous	SRNS	None	Complete remission of NS	Stanczyk et al. (2018)
Japan	0.8	NA	c.782C > T (p.Pro261Leu)	Heterozygous	SRNS	None	Complete remission of proteinuria	Nakanishi et al. (2019)
Turkey	7.0	8.0	c.1058C > A (p.Ala353Asp)	Homozygous	SRNS	None	Kidney function improved with No complications related with the renal transplantation	Yuruk et al. (2020)
Turkey	10	NA	c.1058C > A (p.Ala353Asp)	Homozygous	None	SNHL	Delayed renal or neurological disease	Yuruk et al. (2020)
Turkey	NA	5.0	c.1058C > A (p.Ala353Asp)	Homozygous	SRNS	SNHL, optic atrophy	Visual acuity improved	Justine et al. (2020)
Turkey	4.0	NA	c.1058C > A (p.Ala353Asp)	Homozygous	SRNS	SNHL	Remission of proteinuria; unchanged hearing loss	Justine et al. (2020)

Note: ESRD, end-stage renal disease; NS, nephrotic syndrome; SNHL, sensorineural hearing loss; SRNS, steroid-resistant nephrotic syndrome; ND, not done; NA, not available.

A mutation of the *COQ6* gene was first reported as a cause of primary CoQ10 deficiency in the study of Heeringa et al. (2011) The authors reported that all affected patients (*n* = 11) presenting with SRNS and SNHL showed proteinuria with a median onset age of 1.2 years (range, 0.2–6.4 years) and ESRD with a median onset age of 1.7 years (range, 0.4–9.3 years); five cases also presented with extrarenal involvement such as white matter abnormalities, seizures, ataxia, and facial dysmorphism. Gigante et al. (2017) reported an 8-month-old Italian boy with SRNS that progressed to ESRD when he was 20 months old but with no extra-renal involvements; the pathogenicity of the *COQ6* p.Pro261Leu allele was then confirmed by rescue experiments in yeast. Park et al. (2017) reported six unrelated Korean children with SRNS and hearing loss, and mild muscle weakness of the lower extremities and ocular manifestations including bilateral optic nerve atrophy and exotropia were observed in four patients. The patients developed SRNS with onset at a median onset of 29 (range, 15–47) months and ERSD with onset at a median onset of 45 (range, 17–73) months; five of the patients underwent kidney transplantation without FSGS recurrence. Of note, these above patients with *COQ6* defects manifested SRNS during childhood, and all cases progressed to ERSD within 1–2 years of onset. Furthermore, some affected individuals received timely high-dosage oral CoQ10 administration after *COQ6* mutations were detected, and their renal function and psychomotor development have been improved (Cao et al., 2017; Stanczyk et al., 2018; Justine et al., 2020). Remarkably, patients may not benefit from CoQ10 therapy after severe renal and neurological damages occur. These observations suggest that once COQ10D6 is suspected clinically, immediate CoQ10 treatment and detection of *COQ6* mutations would be helpful for the improvement of patient prognosis.

Notably, clinical heterogeneity has been observed in patients carrying the same *COQ6* mutations (see Table 2). For example, a 7-year-old girl from Turkey showed only SRNS without other systemic symptoms due to a homozygous *COQ6* mutation c.1058C > A (p.Ala353Asp), while her elder brother harboring the same *COQ6* mutation exhibited normal renal functions without any neurological presentations and developed SNHL at 10 years old (Yuruk et al., 2020). Additionally, Heeringa et al. (Heeringa et al., 2011) reported six patients with the *COQ6* variant c.763G > A (p.Gln255Arg) who all had SRNS with or without other extrarenal presentations such as SNHL, seizures, white matter abnormalities, and facial dysmorphism. Similarly, our patients with the same *COQ6* mutations displayed variable clinical presentations, whereby the proband satisfying the diagnostic criteria of NS also showed growth retardation and muscle hypotonia in addition to seizures, which were not present in her older brother. Unlike most cases reported previously, our cases died in early infancy. Our findings highlight the clinical heterogeneity and the severity of the disorder caused by *COQ6* defects.

**FIGURE 4:**
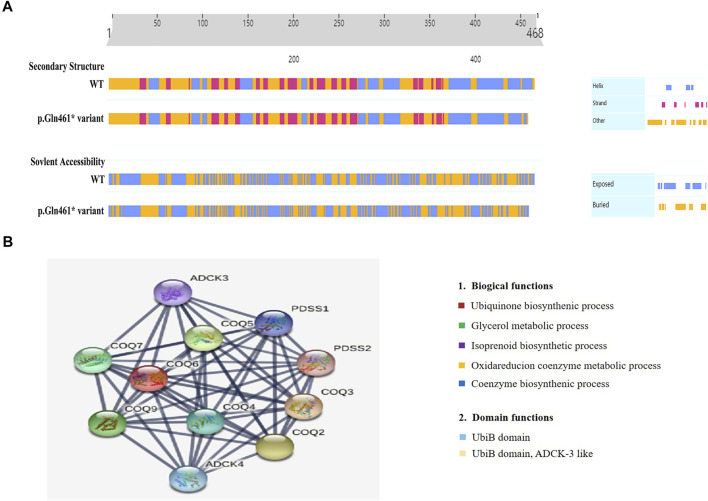
Secondary structure analysis of the *COQ6* protein and STRING network analysis. **(A)** Secondary structure and solvent accessibility of wild-type COQ6 and the protein with the candidate variant (p.Gln461Ter) were analyzed using the PredictProtein online database. **(B)** The functional association of the COQ6 with other proteins involved in the pathway of CoQ10 biosynthesis was predicted by STRING network analysis (STRING v11.0, https://stringdb.org/).

Moreover, we uncovered two novel and rare pathogenic mutations in *COQ6*, which are essential for CoQ10 biosynthesis, and these findings contribute to the known genotypic spectrum of COQ10D6. To date, seven mutations in *COQ6,* including missense mutations, indels, and frameshifts, have been reported in the Human Gene Mutation Database (HGMD). To confirm the deleterious effect of p.Gln461Ter on *COQ6* function, we evaluated the secondary structure of *COQ6* with p.Gln461Ter mutation using PredictProtein and found that it affected the secondary structure and solvent to some extent (Figure 4A). The Gln461Ter led to a deletion of seven amino acids of the *COQ6* C-terminus located on the surface of the active site (Doimo et al., 2014), which was confirmed to be functionally important by a recent study demonstrating that the deletion of 25 amino acids at the *COQ6* C-terminus leads to impaired CoQ10 synthesis (Acosta et al., 2019). These results suggest that the two novel *COQ6* mutants identified in our study are likely both loss-of-function variants; the p.Try81Ter causes NMD and p.Gln461Ter perturbs the active site and secondary structure of the COQ6 protein. In addition, *COQ6* was found to correlate closely with *COQ3*, *COQ4*, *COQ5*, *COQ7*, and *COQ9* in a PPI network (Figure 4B). Human PDSS2, *COQ4*, *COQ6*, and *COQ7* could form a protein complex in human mitochondria, and the knockdown of *COQ6* markedly resulted in reduced levels of *COQ3*; this, in turn, causes an obvious decrease of *COQ7*, which is crucial for maintaining the mitochondrial function (Yen et al., 2020). Furthermore, the decreased expression of the COQ6 protein was revealed to cause kidney damage by inducing mitochondrial dysfunction and apoptosis in podocytes (Song et al., 2018). Thus, we suspect that the candidate pathogenic variants (p.Tyr83Ter and p.Gln461Ter) may impede the interaction between COQ6 and other mitochondrial proteins in the CoQ complex, thus disrupting the pathway of CoQ10 biosynthesis and ultimately impairing renal function. This may be the reason for the more severe phenotype in the current cases.

However, the present study had some limitations. Due to premature mortality in infancy, we could not confirm our molecular finding through the detection of CoQ10 levels in skeletal muscle and the level of COQ6 protein in skin fibroblasts from patients. Although the pathogenicity of the two novel *COQ6* variants identified in our study is explained by several findings, including the clinical phenotype of the two probands overlapping with patients harboring *COQ6* mutations as previously reported, the segregation with the disease, and bioinformatics predictions, in-depth studies are still needed to further determine the impact of the two candidate mutations on *COQ6* gene expression and function.

In conclusion, our study provides the first report of a novel compound heterozygous pathogenic variant of the *COQ6* gene in a Chinese family with severe COQ10D6. Our cases widen the genotypic spectrum of COQ10D6 and highlight the heterogeneity and severity of clinical features associated with *COQ6* mutations. An early diagnosis of *COQ*6 pathogenic mutations in such cases with clinical presentations suggestive of COQ10D6 will facilitate disease diagnosis and therapeutic interventions as well as disease prevention in future generations.

## Materials and Methods

### Ethics Statement

The study was performed in accordance with the Declaration of Helsinki and approved by the Ethics Committee of Taizhou Hospital of Zhejiang Province Affiliated to Wenzhou Medical University. Informed consent was obtained from the family before gene testing.

### Subjects

We collected blood samples and pedigrees after obtaining informed consent from the family. The two affected siblings were diagnosed with infantile NS by pediatric nephrologists based on the age of presentation (4–12 months) and the diagnostic criteria of NS, including serum albumin ≤25 g/L, proteinuria (24 h urine greater than 50 mg/kg/day), and hyperlipidemia (elevated cholesterol and/or triglycerides) (Lombel et al., 2013); (Wang and Greenbaum 2019). The clinical and molecular biological characteristics of the affected individuals were analyzed retrospectively.

### Genetic Testing

Genomic deoxyribonucleic acid was isolated from the patient’s whole blood, and exome sequencing was conducted using an Illumina NovaSeq 6000 sequencing system with 150 bp paired-end reads at an average 100× sequencing depth to cover the maximum genomic variations. The DNA sequence was aligned to the UCSC hg19/GRCh37 reference sequence. Variant calls and annotation were performed with a pipeline based on the Burrows–Wheeler aligner, in-house software, the Genome Aggregation Database (gnomAD), ClinVar, and custom annotation scripts. Thereafter, Sanger sequencing analysis was performed for variant confirmation and segregation using target sequence-specific primers on an ABI 3730xl sequencer and analyzed using Sequencing Analysis Software v5.2 (Applied Biosystems) and SeqMan (DNASTAR) according to the manufacturer’s instructions.

### *In Silico* Analysis

For the evolutionary conservation analysis of *COQ6* candidate variants, the alignment of gene sequences and complete coding sequences from different species were conducted using DNAMAN, including human (NP_872282.1), rhesus monkey (XP_014999576.2), house mouse (NP_766170.2), Norway rat (NP_001011 983.1), and zebrafish (NP_001038869.1). Next, the pathogenicity of the variants was predicted by using MutationTaster, and the possible influence of the detected variants on the structure and function of the COQ6 protein was evaluated by PredictProtein. The diagram of the functional protein association network was depicted based on the STRING (https://string-db.org/) database, which was used to predict protein–protein interactions.

## Data Availability

The datasets for this article are not publicly available due to concerns regarding participant/patient anonymity. Requests to access the datasets should be directed to the corresponding authors.
